# Distinct mechanisms drive post-antibiotic Tuberculosis relapse post-cure versus post-treatment-failure

**DOI:** 10.64898/2026.01.04.697520

**Published:** 2026-01-05

**Authors:** Christian T. Michael, Maral Budak, Philana Ling Lin, Denise Kirschner

**Affiliations:** 1 Kirschner lab, Department of Microbiology & Immunology, University of Michigan - Michigan Medicine, Ann Arbor, MI, USA; 2 Department of Pediatrics, UPMC Children’s Hospital of Pittsburgh, University of Pittsburgh School of Medicine, Pittsburgh, PA, USA

## Abstract

Tuberculosis (TB) remains a global health concern, as *Mycobacterium tuberculosis* (Mtb) infects a quarter of the world’s population. Though many TB patients sterilize infection with treatment regimens including the current standard, incomplete sterilization leads to post-treatment relapse and development of drug resistance. Two mechanisms have been hypothesized as driving relapse: *persistence*, where treatment kills all replicating Mtb, and relapse follows once non-replicating Mtb return to a replicative niche; and *threshold*, where replicating Mtb remain alive, yet below detectable levels. Relapse is often detected through a combination of clinical and bacteriological testing, often clinically described as recurrence of TB <2 years after a “cure” diagnosis, while many experimental studies examine relapse ~2-months after treatment completion. Our capacity to untangle these considerations and identify mechanisms driving relapse *in vivo* are limited. Here, we examine the impact of both threshold and persistence mechanisms on relapse post-treatment completion and post-cure diagnosis using our computational model capturing whole-host Mtb infection dynamics. Simulations show that erroneous TB-negative diagnosis post-treatment (false cure) rates are regimen-specific, specifically, the historic standard HRZE is more likely to result in false cure than the contemporary regimens RMZE or BPaL. We also identify how threshold-driven or persistence-driven relapse correlates with both pre-treatment bacterial burden and diagnostic tests used at treatment completion. Simulations show that post-cure relapse is almost exclusively persistence driven, while threshold-driven relapse is most common without a “cured” inclusion criterion. Thus, for patients with negative bacteriological diagnostic results at treatment completion, subsequent relapse may best be personalized by targeting non-replicating Mtb.

## Importance

Incomplete treatment of TB leads to antibiotic resistance and risks relapse, which may occur years later. Understanding relapse is methodologically challenging but may allow some cases to shorten the 4-month-long recommended treatment timeframe. Predictors of relapse are not well-defined given variability in technical definitions of relapse and nuances of study designs. Here, we simulate both clinical and experimental relapse studies, including multiple diagnostic tests and relapse definitions, using biologically-based computation. We find that two hypothesized types of relapses, each potentially requiring a different treatment strategy, are simultaneously at play. Simulations suggest that relapse after “cure” diagnosis (most clinical studies) is caused by reactivation of non-replicating bacteria hidden from treatment within necrotic granuloma tissue, whereas experiments that classify *any* live Mtb post-treatment as relapse are more likely to report relapse caused by incomplete sterilization of replicating Mtb. Predictions depend on the currently unclear relationship between bacterial burden and clinical symptoms.

## 1. Introduction

Pulmonary tuberculosis (TB) remains a dire concern for countries across the globe. A staggering one-quarter of the human population is infected with *Mycobacterium tuberculosis* (Mtb)^1^, with recently rising rates of infection documented within the United States^2^. Approximately 90% of those infected individuals harbor clinically asymptomatic Latent Tuberculosis Infection (LTBI), while the remainder progress to clinically active TB disease^3–5^. Accurate diagnosis and successful treatment of Mtb infection is required to eradicate TB disease worldwide, as incomplete treatment and adherence issues have led to a rise in drug-resistant Mtb strains^6^. The WHO- and CDC-recommended treatments for drug-susceptible active TB are between 4–9 months^7,8^, and the WHO-recommended treatment for LTBI takes 3–6 months^9^. Human studies suggest that standard treatment results in approximately a 5% relapse rate (i.e. recurrent TB disease after treatment, see Table 1), a number that increases to 20% for patients undergoing short-course treatment^10^. Still, that more than half of TB patients are successfully treated within 2–3 months^11^ presents a critical balancing act for public health agencies and clinicians alike. On one hand, shortening treatment has the potential to reduce the risk of drug-induced side effects, financial and human public health resources. On the other, incomplete treatment risks the possibility of relapse and development of resistance. Confounding this, we lack reliable predictors of post-treatment relapse.

Recent studies have revived a deep interest in the importance of understanding relapse for TB drug treatment^11,18^. A key complicating factor is the presence of granulomas^†^. These complex tumor-like structures are hallmarks of Mtb infection, and they provide infection-site-specific pharmacokinetic and pharmacodynamic barriers to treatment. Mtb trapped within necrotic cores found in most granulomas (i.e. caseum) maintains a non-replicating metabolic state (see Supplemental Material S1)^23^, potentially delaying relapse for long times. Our goal is to revisit the contexts under which relapse occurs and use comparisons between experimental animal models, human datasets and computational modeling to elaborate mechanisms driving relapse and their relationships to clinically-detectable TB treatment outcomes.

### 1.1 Relapse

Clinically, active TB is typically associated with signs (cavitary lesions on chest x-rays, fever, etc.) and symptoms (cough, fatigue) of TB, often with microbiologic confirmation of Mtb infection. During treatment, signs and symptoms often improve (improved chest x-ray, sputum smear and culture are Mtb-negative, resolution of cough and weight loss). Clinical relapse refers to having new signs and symptoms of TB after resolution during treatment when exogenous Mtb re-infection has been ruled out^11^. Table 1 contains detailed terminology for describing TB disease state in the context of treatment.

A recent review^11^ theorized two mutually-compatible mechanisms (referred to there as *concepts*) that may lead to relapse: persistence and threshold. *Persistence* suggests that TB treatment leaves a reservoir of non-replicating Mtb alive in caseum, transitioning the host into a state of LTBI; and/or *a Threshold* where TB treatment leaves a few replicating Mtb alive that are below a level of detection (LOD) until treatment stops and the bacterial niche repopulates. These mechanisms are challenging to separate experimentally and clinically^11^, so there is no definitive answer as to which drives relapse.

If indeed both mechanisms are at play, it is not clear how persistence- and threshold-driven relapse differ in terms of optimal diagnosis or treatment. Relapse is a phenomenological term, typically reported using a composite of one or more measures including positive bacterial smears or cultures, rapid-test molecular analysis, radiographic exams, or clinical TB symptom presentation^11,18,24–26^. Each test for TB cure has advantages and disadvantages (Table 2 and ^11^), although none can categorically predict relapse. Using cynomolgus macaques, a non-human primate (NHP) model of human-like LTBI^27^, it was found that measuring Bronchoalveolar Lavage Fluid (BALF) for CFU reduction over time was insufficient for assessing effective antibiotic treatment, particularly during short-term treatment^28^.

### 1.2 Experimental Models of relapse

**Figure 1:**
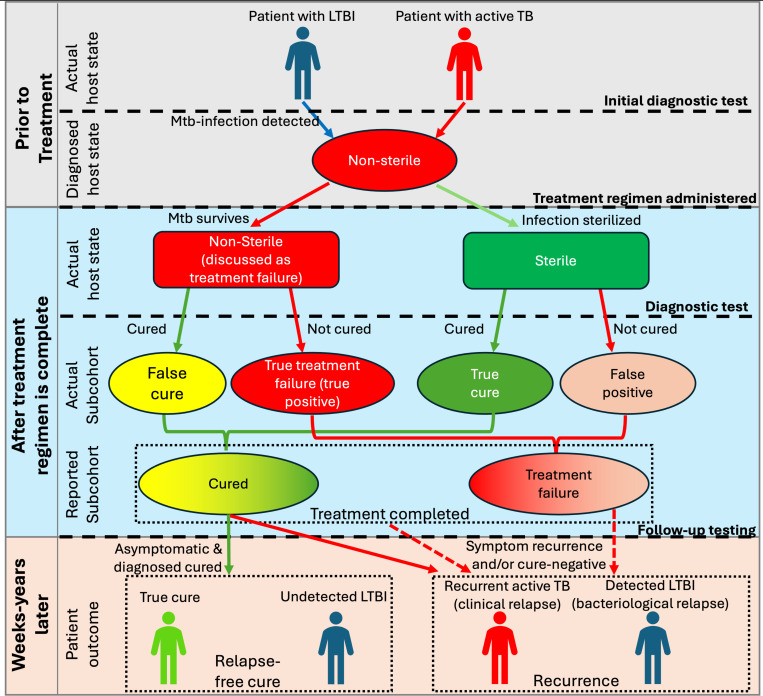
Classifications of TB recurrence and relapse. This flowchart shows the connection of all host states and tests listed in Table 2. Note that this shows three key confounding factors frequently addressed in relapse research. First an overload in discussing treatment success or failure: in clinical trials, conclusions about an actual sterilizing population (third row) must be based on cure reports (fourth row), and “treatment success/failure” is used interchangeably to describe rows 3–5. Second is that clinical studies define relapse to occur after cure, (bottom row, solid arrows) though some studies implicitly include relapse as active TB after treatment completion and/or failure if diagnostic tests are not run upon treatment-completion (bottom row, dashed arrows). Third is the complexity of having interchangeable diagnostic tests at initial diagnosis, treatment completion, and during relapse follow-up. This classification assumes no reinfection (Table 1).

Multiple *in vivo* animal and human studies are employed to study TB outcomes^41^ including relapse, notably including NHP^18,27,42^ and mouse^43–46^ models. Relapse mouse models (RMMs) can be readily treated with human-equivalent doses (i.e., corresponding average plasma concentrations^47,48^) of first and second-line antibiotics. However, most mice do not form necrotic granulomas with the exception of C3HeB/FeJ mice^35,46,49^, and mice have disease states incongruent from those in humans (e.g., no true latent infection). NHPs most closely model human TB, but require expensive resources and expertise^50^. Animal models have been given (intentionally) weak antibiotic regimens to allow for reliable and experimentally-controllable relapse^18^, though this experimental formulation of relapse may be subsequent to treatment completion rather than cure (Figure 1). Moreover, assessing cure in animal models is not always straightforward—repeated sampling of CFU in RMMs is not consistently possible, and such limitations may require testing for relapse post treatment-completion rather than post-cure^14^. Contrasting this, clinical trials are the gold standard for analyzing treatment regimens, but can take years to complete and cost an average of $40M^51^. Testing drug regimens at high risk of TB relapse in humans would be unethical and relapse events in clinical trial datasets are rare and are reported as composites of multiple detection methods^18,24–26^. All clinical relapse studies we examined considered relapse to be post-cure, though relapse was occasionally reported as grouped with treatment failure as “unfavorable outcomes”. Additionally, diagnostic tests used to identify relapse in individual patients was not always reported.

A confounding factor when studying relapse is that there is no directly confirmed connection between the state of a host’s lung infection and their clinical symptoms. Even if one knew the precise number of bacteria and their metabolic state at every timepoint within lungs, we would not know whether culturable or symptomatic relapse will result. It is possible for asymptomatic hosts to shed viable Mtb (subclinical disease), observed in ~5% of nonhuman primates (NHPs), (referred to as percolators^52–54^) as have been described now in humans^55^. Model hosts have also been observed as having levels of inflammation markers such as interferon gamma (IFN-γ), which cannot directly predict host outcomes^56^, highlighting this complication. Previous studies found 10–25% of LTBI patients exhibited neutrophilic whole-blood transcriptomic signatures consistent with active TB^4,57^. Consistent with this, clinical studies have shown multiple inflammatory markers in patients with LTBI^17^. Together, this suggests some LTBI cases involve an ongoing immune siege to contain metabolically-active Mtb from escaping granulomas, while other cases involve surveillance over non-replicating, caseum-trapped Mtb.

There is conflicting terminology between experimental and clinical studies of relapse (Table 1), and it is unclear whether the persistence or threshold mechanisms equivalently underpin distinct notions of relapse. Challenges to experimentally reproducing relapse *in vivo* (e.g., species differing in symptom presentation) necessitate the use of alternative measures than diagnostics used for humans. For instance, CFU enumeration in mouse lung homogenates or NHP granulomas is used as one proxy for clinical relapse (Table 2), even though the detection of small or caseum-bound Mtb populations in such samples may resemble reactivation from latency or recurrent LTBI rather than symptomatic clinical relapse.

To examine mechanisms underpinning various measures of relapse, we extend our virtual host model *HostSim* (see Methods and Supplemental Material S2) to include both persistence and threshold mechanisms as well as virtual diagnostic tests that mimic *in vivo* diagnostics. We use *HostSim* to reproduce both clinical and experimental relapse studies. Our simulations suggest that treatment failure leaves a reservoir of replicating bacteria, leading to rapid threshold-driven relapse (Figure 1). On the other hand, post-cure relapse is primarily persistence-based, i.e., driven by a continuous release of non-replicating bacteria derived from caseum, typically taking much longer between treatment completion and relapse. Simulation results depend on levels of detection of diagnostic tests, many of which are only estimates due to a current lack of understanding of how lung CFU relates to sputum/BALF CFU.

## 2. Results

To predict drivers of relapse, we use our recently-calibrated and validated whole-host TB model, *HostSim*^58–60^. Briefly, *HostSim* is a hybrid agent-based, ordinary differential equation model that represents interaction between host immune cell populations in lungs, uninfected lymph nodes, and blood with Mtb subpopulations within multiple lung granulomas of a virtual host. *HostSim* allows us to simulate temporal trajectories of various T-cell and macrophage population sizes, as well as replicating and non-replicating Mtb within each granuloma of each host (see Methods) and their total in lungs. Recently, we used *HostSim* to compare multiple measurements of drug efficacy of various first- and second-line antibiotic regimens^58^.

For all analyses in this paper, we create a single virtual cohort of 500 Mtb-infected hosts, all inoculated with a single Mtb at 𝑡𝑖𝑚𝑒 = 0. We allow each virtual patient to develop a mature Mtb infection until 300 days p.i. then take a snapshot of its simulation configuration. From those snapshots, we can use one virtual cohort to study multiple *what-if* scenarios wherein we treat the entire cohort with one antibiotic regimen, halt treatment, then observe regimen-specific host and cohort outcomes.

To compare our virtual predictions to both animal and human datasets, we develop a set of virtual TB diagnostics that mimic those currently used (Table 2; Methods). Three key virtual diagnostics are *Virtual Mtb plate, Virtual PET/CT*, and *Virtual clinic score* (Table 3). Given these, we simulate relapse given choices of regimen, diagnostic tests, and follow-up times (Figure 1). For indicated virtual relapse studies, we exclude virtual hosts that are cure-negative at treatment completion. For all virtual relapse studies, we define relapse as a non-cure result at a follow-up time Δ𝑡 after treatment completion. We classify each virtual relapse as persistence-based if, at time of treatment completion, there are 0 replicating CFU within the virtual host and threshold-based otherwise.

### 2.1 Rates of false cure differ between regimens

An essential first step to model any notion of relapse is to capture false cure, i.e. when a non-sterile host is diagnosed as cured at treatment completion (Table 1 and Figure 1). For this analysis, we compare true virtual host sterilization against *Virtual Mtb plate* results, which mimic experimental lung and granuloma homogenate cultures capable of detecting non-replicating bacteria (see Methods and Table 2). We assume there is a LOD of 50 CFU when analyzing the whole-host scale and 10 CFU at the granuloma scale (Methods and ^58,62^). As previously^58^, we also delineate between a *host* that is initially-high-CFU (>total 10,000 CFU pre-treatment) and initially-low-CFU (<total 10,000 CFU pre-treatment), and a *granuloma* that is initially-high-CFU (>1,000 CFU pre-treatment) and initially-low-CFU (<1,000 CFU pre-treatment). It should be noted that high CFU hosts may also have some low CFU granulomas but must have at least one high-CFU granuloma.

**Figure 2.**
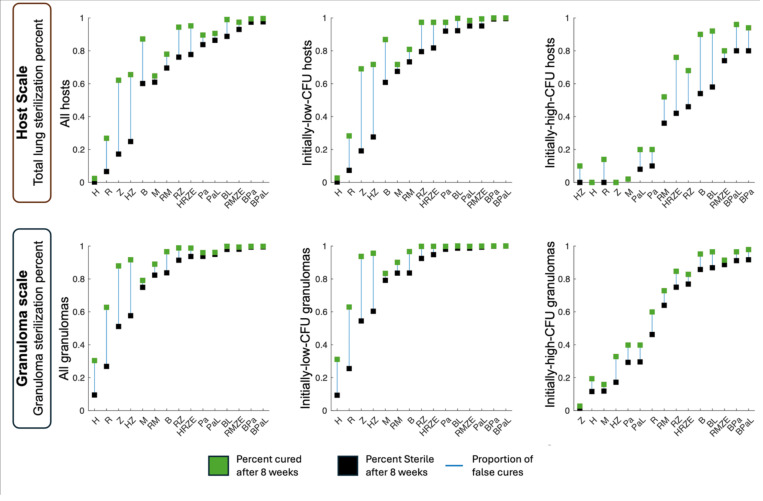
Cure versus sterilization of virtual hosts and granulomas after short-course antibiotic regimens. The virtual cohort containing 500 virtual hosts (shown in top row) and a total of 6500 primary granulomas (shown in bottom row) are each administered a drug regimen for 8 weeks (regimens group (i), see Methods). For each regimen, the percentages for sterilization (black boxes) and lack of detectable infection (green boxes) after 8 weeks of treatment are shown. The left column shows treatment results for each of these virtual hosts and granulomas, whereas the center (right) columns show results for hosts and granulomas that were low-CFU (high-CFU) pre-treatment. We notice that higher-CFU granulomas and hosts are more liable to have subclinical infection (i.e., non-sterile but with CFU < 10 (granuloma) or 50 (host)).

When examining 2-month treatment regimens (group (i) in Methods), we observe a regimen-dependent frequency of false cure at treatment completion at both host and granuloma scales (Figure 2**Error! Reference source not found.**). Our initial observations are not surprising in that most short-course mono-treatments for initially-low-CFU hosts results in large rates of false cure and have little impact on initially-high-CFU hosts. Next, we observe that large granuloma-scale false cure rates amplify into even larger rates at the host scale, as previously predicted^63^. Rates of false cure are increased in initially-high-CFU hosts when compared to initially-low-CFU hosts, particularly after multi-drug regimens, suggesting higher pre-treatment CFU counts are more likely to lead to post-cure relapse. This notably includes HRZE—a first-line standard of care for treating drug-susceptible TB^8^. As *HostSim* is calibrated to recreate treatment of drug-susceptible TB^58^, this suggests that treating high CFU-burden patients with only 8 weeks of HRZE will have a higher risk of false cure (42% sterile vs 76% cured) as compared with regimens including Moxifloxacin or Bedaquiline (e.g., BPaL with 74% sterile, 80% cured and RMZE with 80% sterile and 96% cured). This is consistent with the ~40% relapse rates found for short-course HRZE treatment in a 2016–2018 clinical study^11,64–66^.

We examine the spatial locations of surviving Mtb within false-cured hosts and their non-sterilized granulomas. The location of CFU within granulomas of false-cured hosts depends on their pre-treatment CFU levels, independent of drug regimen. In initially-low-CFU false-cured hosts (middle column of Figure 2), almost all remaining CFU are trapped within caseum. By contrast, in initially-high-CFU false-cured hosts (right column of Figure 2), remaining Mtb are split spatially between trapped in macrophages and caseum. After HRZE treatment, 7/500 virtual hosts are false-cured, and 6 of those 7 are initially-high-CFU. Within the false-cured initially-low-CFU host, nearly all CFU were caseum-bound and non-replicating. One of the six false-cured initially-high-CFU hosts held Mtb intracellularly (inside macrophages), while the others held Mtb within caseum. Pooling HRZE-treated granulomas from all hosts, we observe thirteen virtual granulomas had CFU below LOD—two initially-low-CFU and eleven initially-high-CFU. In those initially-low-CFU granulomas, all undetectable Mtb are trapped within caseum. Of the eleven high-CFU granulomas, eight harbor Mtb solely within caseum while the other three retain Mtb within macrophages at treatment completion. After treating the same virtual cohort with a second-line regimen, BPaL^44,67^, only one initially-high-CFU host holds live CFU below LOD at treatment completion, with all bacteria held entirely within the caseum of an initially-low-CFU granuloma at treatment completion. Overall, these results suggest that when using tests that can detect Mtb within caseum, (i) when clinical relapse occurs in initially-low-CFU hosts, it will likely be persistence-based; and (ii) of relapsing hosts, higher CFU levels pre-treatment are associated with greater chances of threshold-based relapse. Note that these results interrogate the relative likelihood of types of relapse, should it occur, rather than predicting relapse frequency itself events; the NHP study showed that pre-treatment radiographic features did not predict relapse^18^.

Together, these results imply that infection persists in virtual hosts after treatment completion in the form of both false cures and treatment failures (Table 1). Moreover, we have shown that in false-cure cases, Mtb is more likely to be trapped within caseum if the host had low CFU levels prior to treatment, and if a false-cure finds replicating Mtb, such a host had high-CFU prior to treatment. This suggests that if we group relapsing hosts by whether their relapse was persistence-driven versus threshold-driven, those cohorts likely had distinct infection states prior to treatment.

### 2.2 Cure status impacts mechanisms driving virtual relapse in *Virtual PET/CT*

Our current version of *HostSim* captures both persistence and threshold mechanisms that we expect to contribute to post-treatment relapse. First, persistence is a fine-grained representation of non-replicating bacteria stochastically transported out of caseum over time, likely by neutrophils^68^. Such events allow for the possibility of granulomas that harbor CFU within caseum to experience persistence-driven relapse and/or to seed a new granuloma^68^. We calibrate rates of caseum-transport to capture Mtb reactivation of virtual hosts with LTBI during anti-TNF treatment such as etanercept or infliximab^69,70^ (Methods). Secondly, the threshold mechanism is at play when small numbers of live intracellular Mtb can replicate after the end of antibiotic treatment. The threshold mechanism is already present in our model in that intracellular growth of Mtb after treatment is allowable.

Typically, to validate a computational model of, say, relapse, we would generate several virtual hosts and recreate relapse using the two proposed mechanisms above. However, our previous result tells us that the host state prior to treatment must be carefully considered as it likely affects presentation of relapse. Towards defining an appropriate validation dataset, we examine infection states of Mtb hosts from a recent experimental relapse study that employed the use of simian immunodeficiency virus (SIV)^18^. There, NHPs were treated with short-course antibiotic regimens for active-TB only monkeys (8 weeks of isoniazid and rifampicin, i.e. HR). After a one-month rest, they were then infected with SIV to induce relapse^5^. Relapse was primarily measured via observation of newly seeded granulomas using radiolabeled PET/CT scans; the study reported that 8/12 NHPs relapsed. To replicate this experiment using *HostSim*, we represent HIV-1 co-infection as a linear decline of CD4^+^ T-cells calibrated to T-cell blood concentrations reported from the 1990s^71,72^ measured prior to any treatment studies (see Supplemental Material S3). We assume HIV-1 and SIV virtual T-cell depletion behaves similarly^73–76,‡^. We find that many studies report relapse only as a recurrence of active TB disease, possibly with bacteriological follow-up^10,36,77–79^, though detection of non-symptomatic infection after treatment is sometimes reported or discussed as relapse^18,25^ (See bottom row of Figure 1).

Some relapse studies only admit subjects with specific pre-treatment disease states (e.g., active TB), so we similarly predict disease states for virtual hosts. We classify virtual hosts as having active TB if continued bacterial growth is sufficient to cause active disease, which we characterize by a species-specific fold-increase in CFU between treatment completion and follow-up (Methods). In this way, we adjust our classification framework to treat virtual hosts as analogous to humans or NHPs for purposes of disease state prediction. To ensure results are robust to different characterizations of virtual active TB, we also consider virtual hosts with granulomas that individually maintain CFU>1000 for over a month as having active TB.

**Figure 3.**
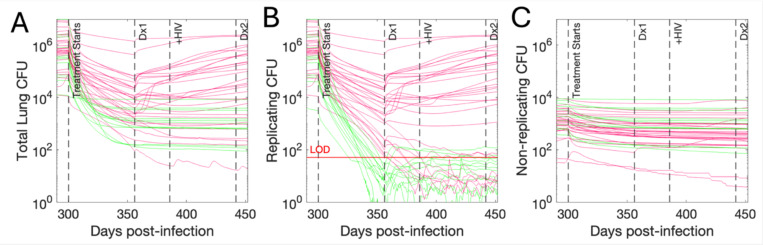
Relapse of virtual hosts after short course of HR and HIV-1 co-infection as in ^18^. Virtual host CFU levels over time for whole-lung total CFU (A), replicating (intracellular + replicating extracellular) Mtb only (B), and non-replicating Mtb only (C). Each line corresponds to a trajectory from one host that was categorized as having active TB prior to receiving HR then relapsing after the end of treatment. Key time points in days post-infection are shown, indicating the start of treatment, the end time of treatment and *Virtual clinic score* diagnostic (Dx1), the onset of HIV, and the time of relapse follow-up diagnostic *Virtual PET/CT* (Dx2). Each curve is colored based on whether the host was assessed as cured at Dx1 (green) or treatment-failed (red) at Dx1. The *Virtual Clinic score* assumes that non-replicating bacteria are not detectable, and the LOD of 50 is shown for replicating CFU.

Virtual disease state classification allows us to examine relapse rates within four subcohorts of our original *n* = 500 virtual hosts, defined by human-like or NHP-like disease state criteria and active/LTBI inclusion criteria, (Table 4). First, we simulate 2 months of HR treatment, at which point we use virtual diagnostics to assess for cure (*Virtual Clinic score*, see Table 3 and Methods), labeling each host as cured or treatment failure at treatment completion. After 4 weeks of drug rest, we simulate virtual SIV infection for a further eight weeks and then assess each virtual host in the full cohort for relapse using *Virtual PET/CT* (Table 3 and Methods) to mimic PET/CT-based relapse NHP observations^18^ (Table 4). We find that the reported percentage of relapsing virtual hosts heavily depends on treatment success or failure (Figure 3). If we only search for relapse in hosts with active TB and reported treatment failure, relapse is nearly guaranteed (>90%). However, approximately 30% of cured hosts relapse within 8 weeks (Figure 3, green curves). The majority of post-cure relapse appears as persistence-driven relapse, mostly exhibiting slowly-increasing levels of intracellular bacteria, consequent to persistence. These relapses tend to have a low, stochastically-oscillating level of Mtb (as bacteria move between niches which is too fine-grained for experimental detection). Consistent with our previous result, more than half of simulated relapse subsequent to treatment failure results from incomplete sterilization of macrophages (Figure 3, red curves), indicating threshold-based relapse. Some of these relapses result in rapid bacterial regrowth in 1–2-week periods that restore CFU to pre-treatment levels. Others exhibit either slow or no regrowth of Mtb populations.

These results confirm expectations set by the previous section when considering *Virtual PET/CT*. Post-cure hosts that relapse are generally low CFU prior to treatment and had primarily persistence-driven relapse, while relapse subsequent to treatment failure appears to be threshold-driven.

### 2.3 Cure status of cohort impacts relapse rates in multiple study designs

We next examine whether our previous observations are preserved across differently-structured relapse studies. For this, we recreate various *in vivo* relapse studies, using virtual diagnostics and subcohorts analogous to those reported *in vivo*. In Table 5, we simulate relapse rates analogous to several studies that (1) report either relapse rates or percent non-cure after standard of care using at least one non-composite diagnostic measure; (2) had varied cohort inclusion criteria and species (including humans, NHPs, and RMMs); and (3) sufficiently indicate whether reported relapse was post-cure or post-treatment-completion. Studies mimicked in Table 5 analyze relapse following many more regimens and timepoints than those recreated here. *HostSim* captures the trend that longer treatment periods are less likely to relapse. In all cases, relapse rates are far higher when including treatment failure in the cohort assessed for relapse.

**Figure 4:**
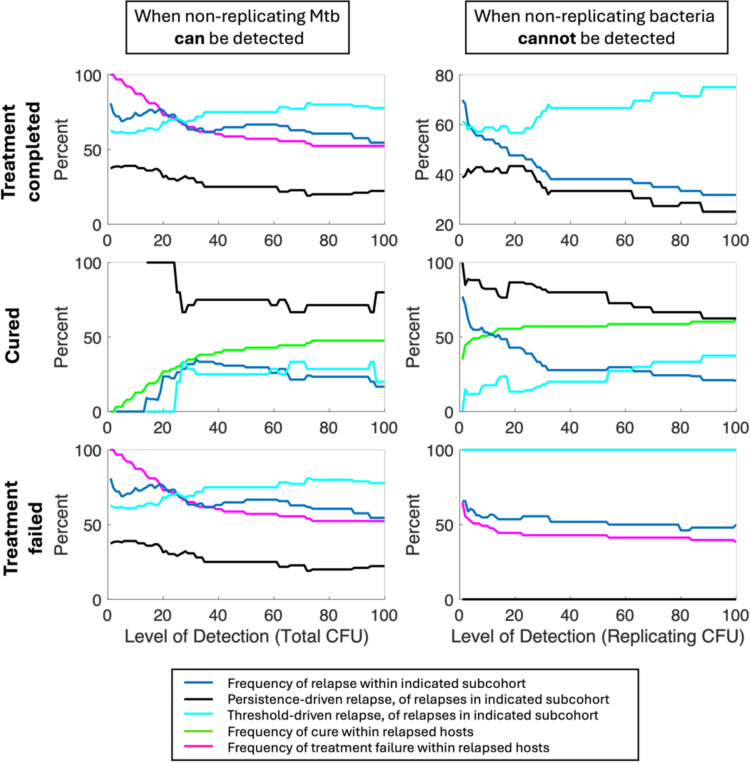
Persistence versus threshold relapse rates by test LOD. (Top row) Each plot shows, for different assumed LODs for *Virtual Mtb plates*, percentage of active TB hosts and relapse events as well as whether those relapse events are persistence-based or threshold-based. (Second and third rows) Portion of the active TB cohort that were cured (middle row) or failed treatment (bottom row), as well as the respective percentages of those cohorts that relapsed, and the percentage of those relapses that are persistence-based. Analyses are repeated assuming the *Virtual Mtb plate* can (left) or cannot (right) detect non-replicating bacteria for diagnosis of cure or relapse.

Lastly, we want to quantify whether reported persistence or threshold-based relapse is affected by sensitivity of diagnostic test used. For this, we examine relapse 1 year after completing a 2-month short-course treatment of HRZE. We assess cure of virtual hosts both at time of treatment completion and one year later by using *Virtual Mtb plate* (Methods), varying the LOD of both initial and follow-up tests from 0 to 100 CFU. We also vary whether or not these diagnostics can detect non-replicating CFU. Figure 4 shows that extremely sensitive tests are unlikely to classify patients as cured, especially if they detect non-replicating bacteria. As tests become less sensitive, more hosts diagnose as cured, and cured hosts are unlikely to relapse. More than half of post-cure relapses are persistence-based. All relapses are more frequently threshold-based if the diagnostic tests do not detect non-replicating bacteria.

## 3. Discussion

Safely shortening antibiotic treatment for TB is a critical step towards eradicating the world’s leading cause of death by infectious disease. Aside from development of resistance, the main threat of shortening treatment regimen administration is relapse, where treated infections later recur. There are likely two modes of relapse: persistence-based (slow, rooted in the transport of non-replicating Mtb out of caseum) and threshold-based (fast, rooted in incomplete sterilization of intracellular-Mtb)^11^. Given the considerable experimental challenges investigating relapse, we explore these phenomena using our whole-host computational model grounded in human and primate datasets.

Using *HostSim*, we compared rates of infection sterilization versus diagnostic cure status after virtual short-course antibiotic treatment regimens, focusing on antibiotics present in standard-of-care treatments such as HRZE^8,81^ (Methods). Simulations suggest that several regimens, including HRZE and most mono-treatments, are far more likely to result in false cure, where non-sterile infection falls below LOD at the time of treatment completion (Figure 2). To investigate outcomes at later times, we develop and calibrate a new persistence mechanism in *HostSim* that allows non-replicating Mtb to be transported from caseum, allowing for new granuloma reseeding and TB relapse. With it, simulations reproduce a recent short-course treatment relapse study in NHPs^18^.

We find that reported relapse rates are considerably impacted by study-specific relapse definition—specifically, whether it is based on (1) cure status upon treatment completion (not standardized between clinical and experimental studies) and (2) whether LTBI patients are admitted to relapse studies (Table 4). This remains true when recreating several other relapse studies in humans, mice, and NHPs. When analyzing datasets as closely as possible to their *in vivo* counterparts, we recapitulate qualitative trends of relapse (e.g., lower relapse rates follow longer treatment regimens). However, we also find a paucity of calibration-relevant measurements in the literature, such as LOD of many diagnostic tests used. Our simulated relapse rates are higher than those reported in literature (Table 5). For human and NHP studies, this is reasonable since virtual diagnostic LODs assume that 1 CFU in the lungs corresponds to 10 CFU/mL in the BAL, amplifying sensitivity. For RMMs, our relapse rates are closer to those observed in caseum-forming C3HeB/FeJ mice than non-caseum-forming BALB/c mice.

For research purposes, recurrent LTBI is seldom discussed in relapse studies because (i) clinical relapse definitions do not typically include recurrent LTBI, and (ii) animal models such as RMMs often use detectable Mtb post-treatment as a proxy for relapse. These proxies may implicitly classify as relapse small amounts of Mtb that more closely resemble recurrent LTBI or reactivation than clinical relapse (Table 1). Further, clinically studying recurrent LTBI would be challenging because, by definition, it is asymptomatic in humans. The absence of such datasets leaves little guidance for what LOD thresholds to use for more detailed simulations. Moreover, regimen-specific risk of recurrent LTBI may be a silent contributor toward drug resistance.

For clinical consideration, a TB patient may be asymptomatic at treatment completion regardless of cure status. However, simulations suggest cure status at treatment completion may predict the mechanism of subsequent relapse, should it arise. Specifically, patients relapsing post-microbiologic-cure are more likely to harbor Mtb reservoirs within caseum rather than residual replicating populations, even if the diagnostic test cannot detect caseum-trapped Mtb (Figure 4). This may inform personalized treatment strategies targeting non-replicating Mtb subpopulations.

We have made several important simplifications to reduce model complexity. We do not model drug resistance, though drug resistance is believed to correlate with relapse rates^10^. Lymph nodes are known to be important reservoirs of Mtb during TB disease and relapse, so we will combine our recent model of lymph node infection into *HostSim* in future work^18,19,21^. Moreover, we calibrate our persistence-like mechanism using reactivation rates subsequent to TNFα depletion (Methods), which may ignore subtleties of TB disease during immunosuppression, evident in how TNFα-induced reactivation of TB still often test skin-test negative, and may have other qualitatively distinct immune factors^11^. Finally, the potential for diagnostic tests to yield false positive results follows from test-specific causes that are beyond the scope of this work. For example, simulating false-positive results from immune assays that likely require detailed representations of markers used in that assay, whereas predicting false positive results from culture-based tests would depend on detailed representation of BALF sampling or laboratory contamination.

There are gaps in literature precluding any comprehensive model of relapse. Our simulation framework (as many others) is modular, allowing us to refine individual components of *HostSim* as necessary. However, our virtual diagnostics rely on a coarse-grain representation—e.g., predicting symptoms via 𝑅, the fold-increase of CFU over 200 days, or assuming direct proportionality between CFU in BALF, sputum and lung tissue. We find such abstractions necessary as biological studies relating host/infection state appear noisy, and it is not mechanistically clear why some hosts with active TB are not culture-positive while some subclinical hosts are culture-negative^4,82^. Once these factors are elucidated experimentally, they may be incorporated into a more comprehensive symptom and diagnostic model of TB to predict relapse. With sufficient mechanism, we could develop *HostSim* into a *digital twin*, a personalized model that uses individual patient datasets to predict the likelihood and mechanism of a specific patient’s relapse. A digital twin predicting relapse mechanism for a given patient could provide decision support for which Mtb subpopulations should be priority targets for the treatment regimen. Similar models have been made for a variety of complex diseases^83–86^.

Computational models serve as repositories that can synthesize large amounts of biological knowledge and clinical data. In this case, we find that relapse, often reported as a single phenomenon, is a combination of at least two major presentations. Our predictions suggest that after treatment failure, relapse is likely to be threshold-driven, appearing rapidly and reproducibly. On the other hand, persistence-driven relapses appear more rarely overall, yet more often within false-cured patients. One observation that is confirmed by our results suggests that if a patient has LTBI (culture-negative TB) at the start of treatment, treatment regimen choice should target non-replicating bacteria towards complete sterilization.

## 4. Methods

Recently, we developed a detailed and complex *in silico* whole-host model of pulmonary Mtb infection and treatment, *HostSim*^58–60,87^ (Section 4.1 and Appendix A). Moreover, *HostSim* contains a whole-host pharmacokinetics (PK) and pharmacodynamics (PD) model, which we used to perform virtual pre-clinical trials to characterize bactericidal activity of various antibiotic regimens^58^.

For all analyses, we generate and calibrated a set of *n* = 500 virtual hosts, each with 13 primary granulomas. These virtual hosts are calibrate and validated as previously described^58^. After calibration, our untreated virtual cohort has 89.2% virtual hosts with LTBI, 0.8% sterilizing, and 10% virtual hosts with active TB disease at 300 days post-infection.

### 4.1 Overview of *HostSim*

Briefly, *HostSim* is a multi-scale hybrid computational model of pulmonary TB, including multiple lung granuloma agents within a single virtual host lung that is coordinated with both a blood compartment and an uninfected virtual lymph node system^58,59^. As pulmonary TB is typically contained to these 3 compartments, we refer to this as a “whole host” for TB. Each virtual granuloma is defined by a set of ordinary differential equations (ODEs) that describe interactions between host immune cells (CD4+ T cells, CD8+ T cells, and macrophages) communicating, polarizing, and differentiating in response to cytokine signals (IFN-γ, TNFα, IL-4, IL-10, and IL-12). These immune cells respond to three distinct Mtb subpopulations: intracellular (within macrophages), extracellular within the granuloma, and non-replicating (trapped within caseum). As Mtb are killed, antigen accumulates and traffics to the lung draining lymph node system, which is represented as another set of ODEs. The lymph node ODE tracks priming of Mtb-specific CD4+ and CD8+ immune cells which migrate to the host’s blood, which is the third and final set of ODEs in *HostSim*. CD4+ and CD8+ Mtb-specific and nonspecific T cells may then be recruited to the virtual lung granulomas based on the granuloma state. This model also has stochastic elements such as granuloma dissemination (i.e., seeding of new granulomas) and the persistence-based mechanism of Mtb transport from caseum to macrophages.

Each ODE term and inter-physiological compartmental transition term describe key dynamics of pulmonary TB (both untreated infection progression and the pharmacokinetics/pharmacodynamics (PK/PD) governing treatment efficacy). The terms in *HostSim* include (i) metabolic differences between replicating Mtb (internalized by macrophages or not) and non-replicating Mtb; (ii) dynamic priming of T cells and the (de)activation of monocyte-derived macrophages; (iii) the dynamic accumulation of caseum based on macrophage necrosis; (iv) antibiotic penetration into caseum; and (v) synergistic/antagonistic drug-drug impacts on PD.

### 4.2 Classifying Virtual Host State

To systematically examine case-specific patient outcomes with or without treatment (Table 1), we need to determine how those outcomes are measured.

#### 4.2.1 Classifying virtual host LTBI versus active TB by species

Estimating which virtual hosts suffer active disease is a nuanced challenge, as there is no currently known mechanism connecting CFU (lung or BALF) to symptoms. As an estimate, if CFU levels reach a species-specific threshold for fold-increase over when we would have expected hosts with LTBI hosts to stabilize (~200 days), then we label the hosts as having active TB. We also predict hosts with large-CFU levels (>10,000 for over 30 day timeframe) also experience active TB, as we have in previous work^58^. See Supplemental Material S4 for more details.

#### 4.2.2 Virtual Diagnostic tests

Clinically or experimentally, disease state is assessed using one or more diagnostics (Table 2). To assess virtual host outcomes, we mimic those detection methods *in silico* using virtual diagnostics (Table 3). These are the building-blocks of testing for more complex outcomes like relapse or reactivation.

A virtual diagnostic test takes a virtual host at a given time and assesses their infection state. All host-scale tests assume that pulmonary TB infection states can be precisely measured by looking at the lung state, but this may be expanded in future studies to include more detailed regarding the role of lymph node infection^21^.

##### Virtual Mtb Plate

This virtual test recreates various culture, smear, or NAAT-based tests (Table 6) to assess a host as either *cured* or *not cured* at a given time. Virtual Mtb plates sum together CFU counts of relevant Mtb bacilli and compare them against a LOD. The test returns *not cured* if the sum of Mtb exceeds the LOD, and it returns *cured* otherwise. The exact definition for which Mtb are relevant is configured by checking a number of biological assumptions; in principle, we can calibrate these further once more biology is known.

##### Virtual PET/CT

Combined positron emission tomography (PET) and computed tomography (CT) is a radiographic measure of inflammation (Supplemental Material S1). As previously^87^, we measure virtual FDG avidity (measured by PET/CT) as a weighted sum of metabolically-active immune cells, including activated macrophages and T cells within each granuloma. In summary, FDG avidity is calculated as

$$\text{Virtual FDG avidity}=w_{1}\text{MR}+w_{2}\text{MI}+w_{3}\text{MA}+w_{4}\text{T}0+w_{5}\text{TE}+w_{6}\text{TEM}$$

where $\langle w_{i}\rangle =\langle 0,5,6,2,4,3\rangle$ are the relative weights of resting, infected, and activated macrophages; and primed, effector, and effector memory cell populations within a granuloma. Resting macrophages have a contribution of 0 as we assume they are at the same level of background activity as the uninvolved lung tissue.

The *Virtual PET/CT* test assesses hosts as relapsed if:

1. Any new granulomas disseminate (Supplemental Material S5).
2. There is >20% increase of virtual FDG avidity between time of treatment completion and time of assessment for any granuloma within the host.

##### Virtual clinic score

Virtual clinic score is a combination of the above tests. A host is determined to be *cured* if none of the following return *not cured*:

1. A *Virtual Mtb plate* with LOD = 16 CFU, assuming that non-replicating bacteria cannot be detected (i.e., *Virtual rapid test,* Table 6).
2. If any new granulomas have formed within the host since the last virtual diagnostic, even below LOD—we assume that this is indicative of symptom recurrence as is assumed in ^18^.

### 4.3 Persistence mechanism - stochastic transport of Mtb from caseum

To simulate both persistence- and threshold-driven relapse, we include persistence and threshold mechanisms in *HostSim*. Simulated persistence allows an activated immune system (such as neutrophils) to transport Mtb from caseum, transitioning them to an intracellular state as suggested by our previous work^68^. We assume that the persistence mechanism is involved in both relapse and reactivation (Table 1). LTBI hosts may go years without reactivation—only 10% of subclinical infection hosts with no comorbidities reactivate at any point in their life^4,15^—so we calibrate our reactivation mechanism for studying relapse model in the context of reactivation induced by TNFα depletion. Details are in Supplemental Material S2.

### 4.4 Simulated Antibiotic Regimens

Relapse has been studied in the context of shortening standard antibiotic regimens, which typically include a combination of Isoniazid (INH; H), Rifampicin (RIF; R), Ethambutol (ETH; E), and Pyrazinamide (PZA; Z), called HRZE^8^. The standard treatment is 6–9 months of HRZE, or 2 months of HRZE and 4 months of HR^88^. Several studies have tested for sputum culture conversion after only 2 months of antibiotic treatment^89^. Second-line regimens for RIF-resistant TB include a combination of Bedaquiline (BDQ; B), Pretomanid (PTM; Pa), Linezolid (LZD; L), and (sometimes) Moxifloxacin (MXF; M), called BPaL(M) ^6,25^.

We simulated two families of antibiotic regimens, summarized in Table 7. To determine variability in the level of non-detectable bacteria across multiple regimens, we reproduce a family of regimens including H, R, Z, E, B, Pa, L, and M tested in our previous work^58^ (group i). We also simulate scenarios wherein virtual hosts are treated with multi-phase regimens (group ii), such as the WHO standard of care (Regimen 2HRZE4HR) that discontinues use of PZA and EMB after two months. After virtual treatments end, we continue simulations for without treatment to examine relapse events, though regimens may be assessed at more than one follow-up time (Table 5).

## Supplementary Material

Supplement 1

Supplement 2

## Figures and Tables

**Table 1.** Definitions of terms used to describe TB assessment and state. We have broadened these definitions to reconcile both clinical terminology and experimental terminology, including distinctions where they may not align. This is partially based on Table 1 from^11^. Many of these entries have no good tests or biomarkers support. We use *host* and *patient* interchangeably, although we use *patient* to encourage clinical consideration and *host* as species-agnostic, also applying to animal models.

*Sterilization (*or *clearance*)	An actual host state wherein all Mtb are dead or nonviable.
*Diagnostic test* (or *diagnostic*)	A test that seeks to classify disease state at a specific time, typically assessing for active TB, LTBI, or cure. The WHO-recommended diagnostic varies based on the age and history of the patient^12^. Many diagnostics also include tests for drug resistance^13^.
*Cure* and *Non-cure* (or *Negative diagnosis* and *Positive diagnosis*)	Potential results of diagnostics test that seek to classify a patient as sterile and/or disease-free after a previously Mtb-positive result (*not to be confused with sterilization*). Results are occasionally presented alongside the general type of assessment used to establish cure, e.g. “microbiological cure”. We use *cure* terminology after prior infection is established.
*Controlled infection*	A non-sterile infection state wherein the total Mtb population within a host or granuloma is prevented from expanding.
*Subclinical infection* (or *subclinical disease/TB*)	A non-sterile infection wherein the patient does not exhibit overt TB disease symptoms yet may shed viable Mtb.
*Latent TB infection* (or *LTBI*)	A non-sterile infection state assumed to be controlled and non-contagious, typically diagnosed by immune or radiological methods^4^.
*Treatment Completion*	A milestone within a host’s regimen wherein the patient has finished the prescribed regimen, independent of cure status.
*Treatment Success*	A disease state wherein a patient is cure-positive at treatment completion.
*Treatment Failure*	A disease state wherein a patient is cure-negative at treatment completion.
*Recurrence*	An instance of diagnosable Mtb infection that had previously been assessed as cured. Recurrence most often describes presentation of active TB disease after cure (*recurrent TB, most common*), but may theoretically describe *recurrent LTBI* (i.e., a situation where a previously-cured patient later is determined to have LTBI.)
*Relapse*	An instance of recurrence after cure (most often and intended^11^) or treatment completion (less common, often due to experimental limitations under the assumption that cure is likely^14^). Relapse can be defined as after either recurrent symptomatic TB (clinical definition) or recurrent LTBI (possible in experiments if only microbiological tests are used and animals do not have human-like symptoms).
*Reactivation*	An instance of active TB after a state of stable LTBI has progressed and infection is no longer controlled^15^.
*Reinfection*	An instance of Mtb infection resulting from a separate exogenous inoculum after a prior Mtb infection, typically after the prior infection is declared cured. Epidemiological factors can make this difficult to distinguish from relapse, requiring molecular markers from both episodes^16^.
*False Cure* *	A *cure* diagnosis while the patient is not sterilized.
*False Positive* *	A *positive* or active TB diagnostic test result while the patient is actually sterilized, e.g. a positive tuberculin skin test on a Mtb-free BCG-vaccinated individual^17^.

* These definitions are not standard terminology but are related to sensitivity and specificity of diagnostic tests; we use these terms because the specific meaning of *positive* (and thus sensitivity/specificity) depends on whether one analyzes *cure positivity* or *Mtb-infection positivity*.

**Table 2:** Common methods for assessing *M. tuberculosis* infection and/or TB disease *in vivo*.

Diagnostic	Advantages	Drawbacks	CFU Level of Detection (LOD)	References
Solid or liquid culture of BAL	Gold standard for positive diagnosis when repeated	Can take weeks to months, requires 2+ tests. Poor predictor of relapse during early infection	1 CFU within BALF or sputum (ideally)	^11,29,30^
WHO-approved rapid diagnostics (e.g., Xpert ultra)	Fast, accurate	Expensive, can bear false positive after previous infection	16 CFU/mL (BALF or sputum)	^31,32^
Acid-fast sputum or BALF smear	Fast, inexpensive	Yields false positives after previous infection, false-positive rate may depend on treatment regimen	~5,000 CFU/mL (BALF or sputum)	^11,33,34^
Whole-lung or tissue homogenate	Highly accurate, detects infections during active TB or LTBI	Animal model only (Performed post-necropsy)	~5CFU/sample	^18(NHP); 35,36(Mouse)^
Radiographic tests (e.g., checking for dissemination using a 18F-fluorodeoxyglucose radio-tagged PET/CT probe)	Non-invasive, spatially resolves individual granuloma disseminations, correlates with Mtb burden, longitudinal data available	Measures inflammation typically associated with bacterial burden but can be host response, which may persist post-cure; unknown precise Mtb LOD	- Not a direct measure of CFU, but FDG avidity from PET/CT is proportional to CFU^37^. (Returns a measure of inflammation in host)	^18,37 (NHP); 38(Human)^
Immune assays	Some, like tuberculin skin tests (TSTs) or interferon gamma release assays (IGRAs), only measure Mtb infection. Standard diagnostic tests include TSTs and QuantiFERON-TB Gold In-Tube (an IGRA).	Unclear as to how long inflammation lasts after Mtb are cleared, not always able to distinguish active TB from LTBI Low quality evidence for the ability to detect LTBI	- Not based on CFU (Returns a positive/negative based on sufficient presence/absence of TB-associated immune markers)	^9,17,39,40^
Recording clinical TB indicators (cough, weight loss, etc.)	Noninvasive. Motivates the patient / care team to test for TB that may be otherwise missed	Insufficient to diagnose TB by themselves	Unclear connection to CFU (Gives clinical picture not linked to infection measures)	^8^

**Table 3:** Virtual diagnosis overview. Each virtual diagnostic test has free parameters adjustable to mimic one or more real diagnostics. See Methods for details.

Diagnostic Test name	Brief description	Free parameters in their calculation
*Virtual Mtb plate*	Evaluating for presence of Mtb derived from a patient sample. Patient sample can be derived from a lung homogenate culture, smear, etc. Descriptions of how we configure *Virtual Mtb plate* to mimic various tests are in Methods. Binary - returns 1 *(not cured*) or 0 (*cured*).	- LOD - Ratio of CFU in sample to CFU in lung (or granuloma) - Whether non-replicating Mtb may be present in virtual sample - Whether or not recently-dead Mtb may be counted towards CFU levels
*Virtual PET/CT*	Examining numbers of metabolically-active immune cells in granulomas allows for the prediction of 18F-fluorodeoxyglucose (FDG) avidity (output of PET/CT scans^61^). Sufficiently increasing FDG avidity indicates a positive diagnosis. Binary - returns 1 *(Positive Increase*) or 0 (*No change*).	- LOD - Fold-increase of FDG-avidity required for a positive diagnosis - Predicted relative contribution of metabolically-active immune cells toward FDG avidity (Methods).
*Virtual clinic score*	Implemented as a combination of the above diagnostics. We assume that if a virtual host’s CFU increase is uncontrolled or if a granuloma disseminates, they will have symptoms and receive a clinical score. Binary - Returns 1 (Either or both of the above are both positive, likely symptomatic), or 0 (Neither of the above are positive, likely asymptomatic).	- All of the parameters of *Virtual Mtb Plate* and *Virtual PET/CT*

**Table 4.** Predicted radiographic relapse rates by subcohort design. Full virtual cohort of 500 are analyzed for relapse using virtual PET/CT testing (see Methods). Relapse rates depend on which virtual hosts are analyzed in a subcohort of interest, analogous to study eligibility criteria based on experimental design. In all rows, we assume active hosts are part of the cohort. Each row shows the relapse rates of virtual hosts by subcohort along with details of how many of those virtual hosts relapse post-TC, post-TF, or post-cure; and whether those relapses are persistence or threshold-based.

Are LTBI hosts included?	Does {CFU > 1000} also mean active?	# hosts that reached TC (full SC) [% relapsed at 8 weeks]	Proportion of P vs T relapse (full SC) %P | %T	# C at TC in SC [% relapsed at 8 weeks]	Proportion of P vs T relapse (post-cure) %P | %T	# TF in SC [% relapsed at 8 weeks]	Proportion of P vs T relapse (post-TF) %P | %T
**NHP-like classification of active/LTBI state**							
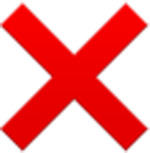	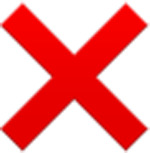	**100 [50%]**	16% P | 84% T	68/100 [29%]	40% P | 60% T	32/100 [93%]	0% P | 100% T
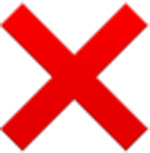	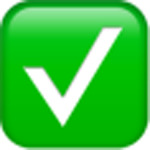	**109 [51%]**	20% P | 80% T	75/109 [32%]	46% P | 54% T	34/109 [94%]	0% P | 100% T
**Human-like classification of active/LTBI state**							
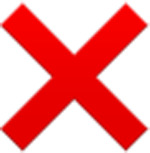	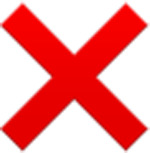	47 [89%]	5% P | 95% T	15/47 [80%]	17% P | 83% T	32/47 [94%]	0% P | 100% T
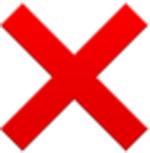	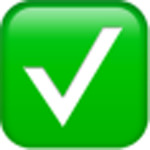	63 [82%]	13% P | 87% T	29/63 [69%]	35% P | 65% T	34/63 [94%]	0% P | 100% T
**Pooling LTBI + active TB (independent of classification-type)**							
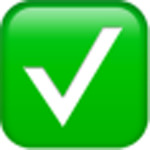	-	483 [17%]	34% P | 66% T	447/483 [11%]	56% P | 44% T	36/483 [88%]	0% P | 100% T

**Bolded** cells indicate simulated results that are the most directly comparable to those from **a recent NHP relapse experiment**^18^**.** SC: Subcohort. TC: Treatment completion. C: Cured. TF: Treatment failed. P: Persistent relapse. T: Threshold relapse.

**Table 5:** Relapse rates from virtual relapse studies that mimic *in vivo* studies. Columns describe several criteria used to configure virtual relapse studies to match an analogous study from literature, with particular emphasis on the selection of the virtual host subcohort for relapse analysis. The full virtual cohort of *n* = 500 hosts are administered antibiotics from Regimen set (ii) (Methods for duration and dosage) and then are simulated without-treatment for a follow-up time before relapse assessment. SC: Subcohort. HC: Homogenate Culture. HS: Host Scale. GS: Granuloma scale. LOD: Level of Detection.

Virtual Relapse Study										Analogous *in vivo* study		
Criteria determining subcohort for relapse analysis							Virtual experiment		Outcome			
Is SC restricted to cure at treatment completion?	Replicated *in vivo* diagnostic test ^A^	LOD in # CFU & test scale	Does test detect non-replicating Mtb?	Does test detect recently-dead Mtb?	Species-parameter used for classifying active TB	SC Size	Treatment regimen	Follow-up time post treatment-completion (Δ*t*)	Virtual Relapse %	Relapse %	Species	Study
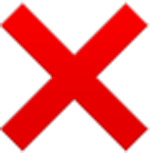	Sputum culture conversion	100^B,C^, HS	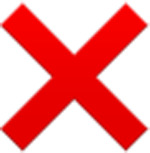	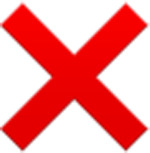	Human	63	2HRZE + 4HR	14 days ^D^	88%	60%	Human	^64–66^
								28 days ^D^	66%	40%		
								60 days ^D^	22%	10%		
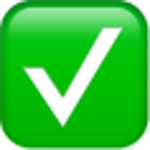	PET/CT inflammation recurrence	1.2x^E^, GS	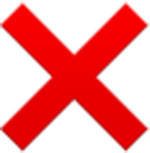 ^B^	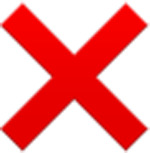	Human	51	2HRZE + 4HR	6 months	43%	12% ^F^	Human	^77^
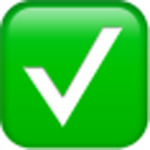 ^G^	Rapid test	16^C^, HS	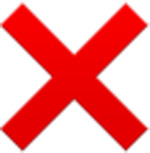	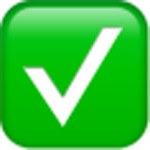	Human	378	2HRZE + 4HR ^H^	2 years	16%	2.2%	Human	80^G^
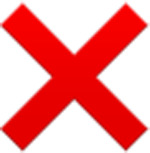	Granuloma Homogenate Culture	10, GS	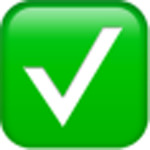	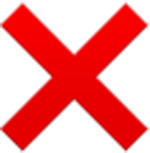	NHP	109	HR with comorbid SIV	8 weeks	100%	72%^I^	Macaque	^18^
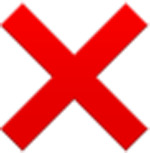	Lung Homogenate Culture	10, HS	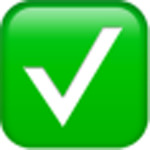	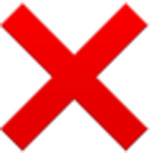	Mouse	104	2RMZ + 2RM	3 months	46%	84%	Outbred Swiss Mouse	^36^
								4 months	50%	42%		
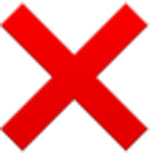	Lung Homogenate culture	10, HS	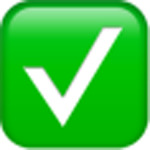	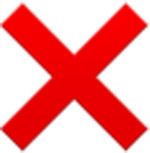	Mouse	105	2HRZ+1HR	3 months	74%	87%	BALB/c Mouse	^45^
								4 months	75%	5%		
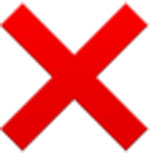	Lung Homogenate Culture	10, HS	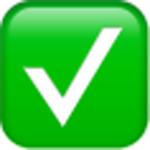	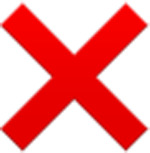	Mouse	104	2HRZE+2HR	3 months	53%	13–27% [0–7%] ^J^	C3HeB/FeJ [BALB/c] Mouse	^46^
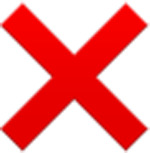	Lung Homogenate Culture	10, HS	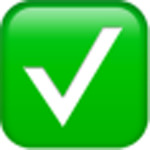	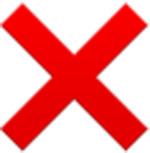	Mouse	104	2RMZE+1RM	3 months	41%	20–60% [0–20%] ^J^	C3HeB/FeJ [BALB/c] Mouse	^46^
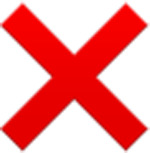	Lung Homogenate Culture	10, HS	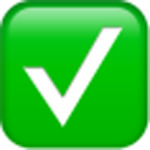	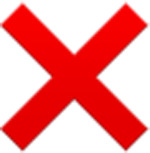	Mouse	104	1BPaMZ+1 BPaM	3 months	48%	7%	BALB/c Mouse	^44^

A – We assume that the same diagnostic test is used for both cure and relapse assessment (Methods).

B – Ansatz.

C – This LOD is the level of detection of CFU for granulomas in *HostSim*, though these reference values come from the CFU/mL in sputum.

D – This did not measure “relapse,” but rather was “still culture positive during continuing treatment” – but was still discussed as potential for relapse in literature^11^.

E – This is a fold-increase of FDG avidity from the time of treatment end, as PET/CT does not directly measure CFU.

F – This is a simplified percentage of 12/99 hosts with recurrent disease post-treatment; actual relationship between PET/CT inflammation and Mtb sterilization is discussed in the original study ^77^.

G – We also include virtual hosts with LTBI, whereas the analogous study includes patients with recurrent active TB after maintaining a long, stable cure state.

H – Actual regimens unknown; we assume that these followed the standard of care.

I – This work did not measure relapse with CFU; this percentage reflects what percentage of NHPs had detectable lung CFU at time of assessment out of all NHPs whose CFU could be counted (See original study ^18^ Fig 3C).

J - These ranges came from experiments using different RMM species, each repeated twice.

**Table 6:** Choices and assumptions that we make to configure virtual Mtb plates into various clinical diagnostics *in silico.* The configuration-based questions are: (1) Does the diagnostic look at the entire host (H) or a specific granuloma tissue (G)? (2) What is the LOD of the test? (3) Are non-replicating or caseum-bound bacteria detectable with this test? (4) Are dead Mtb able to be detected in this test? If so, how long can bacilli be detected for after they have been killed?

Replicated test	Q1 (Scale)	Q2 (What is the LOD?)	Q3 (Are non-replicating Mtb relevant?)	Q4 (Are recently-dead Mtb relevant?)
Sputum or BAL culture	Host	50 CFU^†^	No^†^	No
Acid-fast Mtb smear	Host	5000 CFU	No	Yes, 10 days^†^
Granuloma tissue homogenate Mtb culture	Granuloma	5 CFU	Yes	No
NAAT-based rapid test	Host	16 CFU	No^†^	No^†^,‡
Lung homogenate Mtb culture	Host	10 CFU	Yes	No

† Assume direct proportionality between lung-tissue CFU counts and sputum/BAL CFU counts.

‡ This assumes that the nucleic acid being tested decays rapidly.

**Table 7:** Regimens from previous studies recreated using *HostSim*. This table is partly based on Table 6 from our previous work^58^. Columns labeled by antibiotic name (e.g., INH) indicate the human-equivalent dosage of that antibiotic in human-equivalent mg/kg, administered daily. Each regimen in set (i) lasts 6 months (180 days). Regimens in set (ii) are split into phases, with each phase duration and the drugs administered during that phase indicated by regimen name—e.g., 2HR1R indicates 2 months of INH+RIF followed by 1 month of RIF monotreatment.

Regimen Name	INH	RIF	PZA	EMB	BDQ	PTM	LZD	MXF
**Regimen set (i)** - Marmoset studies from a previous *in silico* / NHP study^90^ (set reproduced from our previous *HostSim* study^58^).								
RMZE	-	10	25	20	-	-	-	7
BPa	-	-	-	-	20	20	-	-
BPaL	-	-	-	-	20	20	90	-
BL	-	-	-	-	20	-	90	-
RM	-	10	-	-	-	-	-	7
HRZE	5	10	25	20	-	-	-	-
PaL	-	-	-	-	-	20	90	-
Bedaquiline	-	-	-	-	20	-	-	-
Pretomanid	-	-	-	-	-	20	-	-
RZ	-	10	25	-	-	-	-	-
HZ	5	-	25	-	-	-	-	-
Moxifloxacin	-	-	-	-	-	-	-	7
Pyrazinamide	-	-	25	-	-	-	-	-
Rifampicin	-	10	-	-	-	-	-	-
Isoniazid	5	-	-	-	-	-	-	-
**Regimen set (ii)** - Multi-phase regimens used in standard of care and/or experimental relapse studies.								
2HR + SIV^‡^	6	10	-	-	-	-	-	-
2HRZE4HR	6	10	25*	20*	-	-	-	-
2RMZ2RM^§^ (^36^)	-	10	25*	-	-	-	-	7
2HRZE2HR^§^ (^46^)	5	10	25*	20*	-	-	-	-
2RMZE1RM^§^ (^46^)	-	10	25*	20*	-	-	-	7
2HRZ1HR^§^ (^45^)	6	10	25*	-	-	-	-	-
1BPaMZ1BPaM^§^ (^44^)	-	-	25*	-	20	20	-	7

* Antibiotic halted after initial phase.

† Culture positivity was reported during treatment, so we predicted relapse at days-post-treatment-start for these specific regimens and times.

‡ Specific timing of virtual HRZE and virtual SIV are given in the Results text, and are adapted from an NHP model^18^.

§ Dose adjusted to the human equivalent standard dose.

## Footnotes

† Note that we refer to lung granulomas unless otherwise specified, though granulomas commonly form in thoracic lymph nodes^19^, likely impacting adaptive immune priming^20,21^; granulomas may also form during extrapulmonary TB—e.g., liver^22^.

‡ Note that (1) HIV-1 and SIV are not identical - e.g., SIV rarely causing AIDS-like symptoms in natural hosts^73^; and (2) there is evidence that SIV-induced relapse is not identical to CD4+ T cell depletion-induced relapse^74^.
